# Factors affecting the knowledge levels, awareness, attitudes, and behaviors of family physicians about child abuse and neglect in Turkiye and changes in them with the training provided

**DOI:** 10.3389/fpubh.2025.1581720

**Published:** 2025-06-19

**Authors:** Muhammet Bayraktar, Serdar Deniz, Elçin Balcı, Rifat Güveli, Abdullah Bircan

**Affiliations:** ^1^Department of Public Health, Faculty of Medicine, Niğde Ömer Halisdemir University, Niğde, Türkiye; ^2^Department of Public Health, Faculty of Medicine, Malatya Turgut Özal University, Malatya, Türkiye; ^3^Department of Public Health, Faculty of Medicine, Erciyes University, Kayseri, Türkiye; ^4^Provincial Health Directorate, Family Health Center, Niğde, Türkiye

**Keywords:** child abuse, child neglect, family physician (FP), in-service training, legal liabilities

## Abstract

**Introduction:**

This study aimed to determine family physicians’ attitudes, behaviors, and levels of knowledge about child abuse and neglect (CAaN) and examine changes through in-service training provided by academics who have studies on the subject.

**Materials and methods:**

This cross-sectional and intervention-type study was conducted between September and December 2017 with the required permissions taken. All family physicians working in 116 family medicine units in family health centers in Niğde province were included in the study. It was conducted in two stages, using survey forms before and after the education given to the participants. The study was completed with 96 family physicians.

**Results:**

The mean age of the physicians participating in the study was 40.2 ± 7.84 years. Thirty-five (36.5%) were female, 85 (88.5%) were married, and 75 (78.1%) had children. They have been practicing medicine for 15.1 ± 7.81 years, and 90 (93.8%) are general practitioners. Eighty-eight (91.7%) expressed that they give special importance to signs of neglect and abuse in children brought in for examination. Thirty-one (32.3%) stated that they have encountered signs of neglect and/ or abuse in children during their professional lives, and 8 (8.3%) have encountered signs of abuse and/or neglect in the last year. Fifty-five (57.3%) received training on CAaN during their education at medical school, and 72 (75.0%) received in-service training on CAaN after they started working as physicians. Eighty-two (85.4%) stated that if training were to be organized on CAaN, would want to attend to receive information on the subject and increase/ renew their knowledge, and 88 (91.7%) stated that they would like to be informed about the results of the study. The changes in the scale subcategories’ mean scores and the general mean scores of the scale in the pre-test administered before the training and the post-test administered after the training show that the participants had a development between the two applications to have sufficient knowledge about the subject with the training, statistically significantly (*p* < 0.05).

**Conclusion:**

In-service training given to family physicians has positive effects on the detection of CAaN. Awareness and knowledge levels should be maintained with regular in-service trainings.

## 1 Introduction

One of the fundamental problems of today’s societies is the phenomenon of violence. Cultural, social, and economic factors have roles in the formation of violence, which is seen as a behavior that exists instinctively and originates from environmental factors. Active and passive actions of parents that prevent children’s development can harm children in the form of child abuse and neglect (CAaN). As such, it is considered a considerable element of domestic violence (1).

Family health centers are designed as the first points of contact for individuals with the health system in Türkiye. Family physicians working in these centers have a special role in the health of society: They are expected to address health problems in terms of physical, psychological, social, cultural, and existential dimensions (2). Although they also need to be competent in recognizing and evaluating signs of abuse and neglect in patients who apply to them for various reasons due to this responsibility, various previous studies have shown that primary health care workers generally lack knowledge, awareness, strategies, and need training regarding multiple forms of CAaN (3–6). In addition, if they fail to identify a child in question in terms of neglect and abuse and fail to fulfill their obligation to report this situation to the relevant institutions, they will have failed to fulfill their legal obligations (6, 7). Per the Child Protection Law in Türkiye, health institutions, like other official institutions and organizations, are also obliged to report children in need of protection to the relevant official institution (8). In addition, family physicians, just like healthcare professionals such as physicians of all specializations, dentists, pharmacists, midwives, nurses, and et cetera, are obliged to report any signs of crime they encounter while performing their duties, following article 280 of the Turkish Penal Code. Therefore, CAaN should also be evaluated and hence have to be reported by them within this scope (9).

This study aimed to determine the awareness levels and attitudes of family physicians toward CAaN, their behaviors when they encounter a neglected/ abused child, and their levels of knowledge regarding diagnosing neglect/ abuse and what should be done when faced with such a situation. It was also aimed to examine the changes in these after an intervention in the form of an in-service training provided by academics who have conducted previous studies on the CAaN topic.

## 2 Materials and methods

This study, which was both cross-sectional and intervention-type, was conducted between September and December 2017 with the approval of the Clinical Research Ethics Committee of Erciyes University numbered 2017/224 and the permission of the General Directorate of Public Health.

All family physicians working in 116 family medicine units in family health centers in Niğde province were included in the study and no additional sample was determined. This study, in which 100 family physicians (86.2% of the universe) working under contract or on assignment in family health centers throughout the province agreed to participate, was conducted in two stages:

- In the first part of the study; after the individuals were informed about the survey forms and their verbal consent for participation was obtained, the survey forms including the informed voluntary consent were delivered to the participants and the survey forms filled out by the participants were received by the researchers.
- In the second part; the participants were trained by academics who have studies on the subject and the surveys were repeated. In this section, four family physicians could not continue working for various reasons and the study was completed with 96 family physicians, which constituted 82.8% of the universe.

Considering the sections where the participants received low scores on the scale applied in the first part of the study, a team all of whom are medical doctors and have studies in the field of public health, led by an academic who is a professor at a medical school, a medical doctor and a public health specialist, and who has studies about CAaN topic, gave theoretical training on CAaN findings to the participants in groups, lasting 1 day, and the scale was re-applied within 2 weeks after this training.

For collecting the data; the “Participant Information Form” consisting of 18 questions, integrated by researchers in line with the literature information by taking as an example the survey forms previously used in similar studies, and used to understand the characteristics of the participants and their experiences and thoughts on the subject, and “Scale Form for Diagnosing the Symptoms and Risks of Child Abuse and Neglect” formed from 67 questions, developed and used by Uysal (10), whose validity and reliability checks have been carried out and whose value of Cronbach alpha was 0.92, were used.

The score values for 46 questions from the 67 in the scale form are as follows; very true = 5, quite right = 4, I am undecided = 3, not quite right = 2, and not true at all = 1 point. The score values for the remaining 21 questions are the opposite: very true = 1, quite right = 2, I am undecided = 3, not quite right = 4, not true at all = 5. The formula used for the calculation of the average score for the subcategories of physical signs of abuse (PS), signs of neglect (SN), behavioral signs of abuse and neglect (BS), parental characteristics (PC), child characteristics (CC), family characteristics (FC) and the overall scale (OS) consisting of these questions is as follows: score = (sum of scores in the relevant category/number of questions in the relevant category), and therefore the lowest average score is one, while the highest average score is five. In other words, scores above three can be interpreted as sufficient knowledge of the subject, and scores below three can be interpreted as insufficient knowledge of the subject.

The data were evaluated in a computer environment using SPSS program version 22. The data’s conformity to normal distribution was tested by the skewness and kurtosis values (11) along with the Kolmogorov–Smirnov test. The means are shown with standard deviations, and the numbers are shown with percentage values. In cases where parametric assumptions are met; the differences between the means of two independent groups were evaluated with the t-test in independent samples and the differences between the means of two dependent groups were evaluated with the t-test of paired samples, while the Mann–Whitney U and Wilcoxon signed ranks tests were used as non-parametric alternative tests. The level of significance was determined statistically as *p* < 0.05.

In cases where the difference between pre-training and post-training test scores was found to be significant, the effect size value was also calculated with Cohen’s d value to show the extent of the difference between the means of the groups. The calculated Cohen’s d value was interpreted relatively as according to the suggestion that d = 0.2 be considered a “small” effect size, 0.5 represents a “medium” effect size, and 0.8 a “large” effect size (12).

## 3 Results

The mean age of the physicians participating in the study was 40.2 ± 7.84 years, 35 (36.5%) were female, 85 (88.5%) were married, and 75 (78.1%) had children. The participants’ descriptive characteristics are shown in Table 1.

**Table 1:** The participants’ descriptive characteristics, professional experience and status of receiving training on the subject.

Variable	Groups	n (%)	x ¯ ± SD
Age			40.2 ± 7.84
Gender	Female	35 (36.5%)	
	Male	61 (63.5%)	
Marital status	Married	85 (88.5%)	
	Single	11 (11.5%)	
Child status	Have at least a child	75 (78.1%)	
	Have no children	21 (21.9%)	
The duration of their medical practice			15.1 ± 7.81
Specialty in medicine	General practitioner	90 (93.8%)	
	Family medicine specialist	6 (6.2%)	
Pay special attention to CAaN symptoms	Yes	88 (91.7%)	
	No	8 (8.3%)	
CAaN finding during the professional life	Yes	31 (32.3%)	
	No	65 (67.7%)	
CAaN findings in the last year	Yes	8 (8.3%)	
	No	88 (91.7%)	
CAaN training at the faculty	Yes	55 (57.3%)	
	No	41 (42.7%)	
Post-graduation CAaN training	Yes	72 (75.0%)	
	No	24 (25.0%)	
Request to participate in training	Yes	82 (85.4%)	
	No	14 (14.6%)	
Information on study results	Yes	88 (91.7%)	
	No	8 (8.3%)	

n, number; %, percentage rate; x̄, arithmetic mean; SD, standard deviation.

The average length of time they have been practicing medicine is 15.1 ± 7.81 years, and 90 (93.8%) of them are general practitioners. 88 (91.7%) of them expressed that they give special importance to signs of neglect and abuse in children brought in for examination, and 31 (32.3%) of them stated that they have encountered signs of neglect and/ or abuse in children during their professional lives, and 8 (8.3%) of them have encountered signs of abuse and/or neglect in the last year. 55 (57.3%) of them received training on CAaN during their education at medical school, and 72 (75.0%) of them received in-service training on CAaN after they started working as physicians. 82 (85.4%) of the participants stated that if training were to be organized on CAaN, they would want to attend to receive information on the subject and increase/ renew their knowledge, and 88 (91.7%) of them stated that they would like to be informed about the results of the study. Participants’ professional experiences and their training on this subject are presented in Table 1.

The situations stated by the participants as reasons for hesitation when reporting CAaN are listed in Table 2, from most to least.

**Table 2:** Reasons for hesitation of participants while reporting CAaN.

Reasons	n (%)
Thinking that the pertinent officialdom will not take necessary action	65 (67.7%)
Worrying about being worn out, pressured, and threatened in processes such as police stations, courts, etc.	56 (58.3%)
Not knowing that a report should be placed or how to place a report.	43 (44.8%)
Wanting not to be involved in an unpleasant issue.	42 (43.8%)
Thinking that the child’s life will be negatively affected after the report.	37 (38.5%)
Not suspecting abuse or neglect.	37 (38.5%)

n, number; %, percentage rate.

In case a training is organized on the subject, the topics that the participants want to be in the training are listed in Table 3 according to the rate of demand.

**Table 3:** Topics that participants would like to see in a training session.

Subject headings	n (%)
Information on what to do if child neglect and/or abuse is detected.	71 (74.0%)
Particulars on directives within the law relating to reporting child neglect and/ or abuse.	71 (74.0%)
Particulars on symptoms and signs of child neglect and abuse.	70 (72.9%)
Information on the responsibilities and obligations of health personnel on the subject.	59 (61.5%)
Information on the subsequent lives of children whose getting neglected and/or abused has been reported to official authorities.	56 (58.3%)

n, number; %, percentage rate.

When the changes in the scale subcategories’ mean scores and the general mean scores of the scale in the pre-test administered before the training and the post-test administered after the training are examined in the study group (Table 4), it is seen that the participants showed a development between the two applications to have sufficient knowledge about the subject with the training, and this situation is statistically significant (*p* < 0.05).

**Table 4:** Mean scores’ comparisons before and after training.

Scale dimensions	Test time	x ¯ (95% CI) ± SD	Δ x̄ (95% CI) ± SD	*p* ^*^	Cohen’s d
Physical signs of abuse	post-test	4.00 (3.91–4.09) **±** 0.44	2.21 (2.08–2.33) **±** 0.63	***<0.001***	3.51
	pre-test	1.80 (1.70–1.89) **±** 0.50			
Signs of neglect	post-test	3.93 (3.81–4.05) **±** 0.57	2.29 (2.11–2.46) **±** 0.85	***<0.001***	2.69
	pre-test	1.64 (1.52–1.77) **±** 0.63			
Behavioral signs of abuse and neglect	post-test	3.87 (3.78–3.95) **±** 0.42	1.86 (1.72–2.00) **±** 0.69	***<0.001***	2.70
	pre-test	2.01 (1.91–2.11) **±** 0.51			
Parental characteristics	post-test	3.64 (3.55–3.73) **±** 0.44	1.62 (1.49–1.76) **±** 0.67	***<0.001***	2.42
	pre-test	2.02 (1.90–2.13) **±** 0.57			
Child characteristics	post-test	3.30 (3.19–3.41) **±** 0.54	0.85 (0.68–1.02) **±** 0.85	***<0.001***	1.00
	pre-test	2.45 (2.32–2.58) **±** 0.65			
Familial characteristics	post-test	3.81 (3.69–3.93) **±** 0.60	1.80 (1.62–1.99) **±** 0.89	***<0.001***	2.02
	pre-test	2.00 (1.87–2.14) **±** 0.66			
Overall scale	post-test	3.81 (3.74–3.89) **±** 0.37	1.86 (1.75–1.98) **±** 0.57	***<0.001***	3.26
	pre-test	1.95 (1.86–2.05) **±** 0.47			

$\bar{x}$ , arithmetic mean; SD, standard deviation; Δ, difference; CI, confidence interval; *paired samples *t*-test. Values with bolded fonts are statistically significant.

As can be seen from Table 5, which shows the pre and post education values of the scale general and subcategory mean scores and their relationship with the participants’ descriptive characteristics, professional experiences, and status of receiving training on the subject; there is an increase in the post-education post-test scores compared to the participants’ pre-test results. Except for the average scores of the CC subcategory for family medicine specialists, participants who were not particularly concentrate to signs of neglect and abuse in children brought in for examination, and those who stated that they did not want to be informed about the results obtained as a result of this study (*p* > 0.05), the increase in the OS and subcategory average scores of all other groups is statistically significant (*p* < 0.05). In addition, while there was not a significant difference in the pre-test mean scores (*p* > 0.05), the post-test SN sub-category mean score was statistically significantly higher in women than in men (*p* < 0.05).

**Table 5:** Mean scale scores of the participants according to their descriptive characteristics, professional experience and educational status.

Variable	Groups	n (%)	PS-pre	PS-post	*p*	SN-pre	SN-post	*p*	BS-pre	BS-post	*p*	PC-pre	PC-post	*p*	CC-pre	CC-post	*p*	FC-pre	FC-post	p	OS-pre	OS-post	*p*
Gender	Female	35 (% 36.5)	1.73 ± 0.47	4.04 ± 0.44	***<0.001***^**a**^	1.63 ± 0.60	4.09 ± 0.56	***<0.001***^**a**^	2.03 ± 0.48	3.87 ± 0.41	***<0.001***^**a**^	1.88 ± 0.59	3.57 ± 0.53	***<0.001***^**a**^	2.42 ± 0.55	3.28 ± 0.53	***<0.001*** ^**a**^	1.94 ± 0.63	3.86 ± 0.68	***<0.001***^**a**^	1.90 ± 0.44	3.83 ± 0.41	***<0.001***^**a**^
	Male	61 (% 63.5)	1.83 ± 0.50	3.98 ± 0.44	***<0.001***^**a**^	1.65 ± 0.65	3.84 ± 0.56	***<0.001***^**a**^	2.00 ± 0.53	3.87 ± 0.44	***<0.001***^**a**^	2.10 ± 0.56	3.68 ± 0.37	***<0.001***^**a**^	2.47 ± 0.70	3.31 ± 0.55	***<0.001***^**a**^	2.04 ± 0.68	3.78 ± 0.56	***<0.001***^**a**^	1.98 ± 0.49	3.80 ± 0.35	***<0.001***^**a**^
		**p**^**b**^	*0.313*	*0.549*		*0.851*	***0.043***		*0.771*	*0.940*		*0.073*	*0.293*		*0.753*	*0.740*		*0.470*	*0.497*		*0.419*	*0.695*	
Marital status	Married	85 (% 88.5)	1.79 ± 0.49	3.99 ± 0.45	***<0.001***^**a**^	1.62 ± 0.61	3.95 ± 0.57	***<0.001***^**a**^	1.97 ± 0.51	3.86 ± 0.42	***<0.001***^**a**^	1.99 ± 0.58	3.63 ± 0.45	***<0.001***^**a**^	2.42 ± 0.67	3.30 ± 0.55	***<0.001***^**a**^	1.97 ± 0.66	3.82 ± 0.62	***<0.001***^**a**^	1.93 ± 0.47	3.81 ± 0.37	***<0.001***^**a**^
	Single	11 (% 11.5)	1.81 ± 0.50	4.08 ± 0.35	**0.003**^***d***^	1.81 ± 0.80	3.74 ± 0.61	***0.003***^***d***^	2.33 ± 0.41	3.91 ± 0.46	***0.003***^***d***^	2.27 ± 0.47	3.70 ± 0.36	***0.003***^***d***^	2.67 ± 0.41	3.35 ± 0.44	***0.010***^***d***^	2.28 ± 0.57	3.75 ± 0.44	***0.003***^***d***^	2.14 ± 0.42	3.83 ± 0.34	***0.003***^***d***^
		**p**^**c**^	*0.678*	*0.527*		*0.575*	*0.390*		***0.024***	*0.695*		*0.177*	*0.511*		*0.208*	*0.664*		*0.126*	*0.795*		*0.114*	*0.895*	
Child status	He has a child	75 (% 78.1)	1.80 ± 0.50	3.96 ± 0.45	***<0.001***^**a**^	1.65 ± 0.60	3.91 ± 0.58	***<0.001***^**a**^	1.96 ± 0.51	3.83 ± 0.44	***<0.001***^**a**^	2.00 ± 0.59	3.61 ± 0.45	***<0.001***^**a**^	2.41 ± 0.69	3.29 ± 0.57	***<0.001***^**a**^	1.95 ± 0.66	3.79 ± 0.64	***<0.001***^**a**^	1.93 ± 0.48	3.78 ± 0.38	***<0.001***^**a**^
	He has no children	21 (% 21.9)	1.78 ± 0.47	4.15 ± 0.38	***<0.001***^**a**^	1.63 ± 0.74	4.01 ± 0.57	***<0.001***^**a**^	2.19 ± 0.47	4.01 ± 0.31	***<0.001***^**a**^	2.10 ± 0.53	3.74 ± 0.35	***<0.001***^**a**^	2.60 ± 0.46	3.35 ± 0.39	***<0.001***^**a**^	2.18 ± 0.65	3.89 ± 0.47	***<0.001***^**a**^	2.03 ± 0.44	3.93 ± 0.30	***<0.001***^**a**^
		**p**^**b**^	*0.850*	*0.086*		*0.924*	*0.453*		*0.061*	***0.039***		*0.485*	*0.244*		*0.156*	*0.567*		*0.156*	*0.472*		*0.360*	*0.110*	
Title	General practitioner	90 (93.8)	1.76 ± 0.48	3.99 ± 0.44	***<0.001***^**a**^	1.61 ± 0.57	3.93 ± 0.58	***<0.001***^**a**^	1.99 ± 0.50	3.86 ± 0.42	******<0.001***^**a**^***	1.99 ± 0.58	3.64 ± 0.44	***<0.001***^**a**^	2.43 ± 0.66	3.30 ± 0.54	***<0.001***^**a**^	1.97 ± 0.66	3.80 ± 0.59	***<0.001***^**a**^	1.93 ± 0.46	3.81 ± 0.37	***<0.001***^**a**^
	Family medicine specialist	6 (% 6.2)	2.27 ± 0.52	4.11 ± 0.47	***0.028***^***d***^	2.05 ± 1.20	3.98 ± 0.52	***0.028***^***d***^	2.26 ± 0.54	4.03 ± 0.54	***0.028***^***d***^	2.44 ± 0.32	3.57 ± 0.40	***0.028***^***d***^	2.75 ± 0.31	3.25 ± 0.50	*0.115*^***d***^	2.46 ± 0.41	3.88 ± 0.84	***0.046***^***d***^	2.34 ± 0.47	3.88 ± 0.43	***0.028*** ^***d***^
		**p**^**c**^	***0.026***	*0.596*		*0.623*	*0.933*		*0.325*	*0.524*		*0.053*	*0.756*		*0.221*	*0.903*		*0.086*	*0.739*		***0.047***	*0.639*	
Pay special attention to CAaN symptoms	Yes	88 (91.7%)	1.80 ± 0.50	4.01 ± 0.44	***<0.001***^**a**^	1.65 ± 0.64	3.94 ± 0.57	***<0.001***^**a**^	2.01 ± 0.51	3.90 ± 0.41	***<0.001***^**a**^	2.03 ± 0.59	3.66 ± 0.42	***<0.001***^**a**^	2.45 ± 0.65	3.31 ± 0.54	***<0.001***^**a**^	1.98 ± 0.67	3.83 ± 0.60	***<0.001***^**a**^	1.95 ± 0.48	3.83 ± 0.36	***<0.001***^**a**^
	No	8 (% 8.3)	1.78 ± 0.42	3.91 ± 0.42	***0.012***^***d***^	1.63 ± 0.60	3.82 ± 0.65	******0.012***^***d***^***	1.98 ± 0.51	3.54 ± 0.51	***0.012***^***d***^	1.92 ± 0.47	3.44 ± 0.60	***0.012***^***d***^	2.50 ± 0.68	3.17 ± 0.53	*0.141*^***d***^	2.25 ± 0.50	3.55 ± 0.56	***0.012***^***d***^	1.95 ± 0.38	3.63 ± 0.43	***0.012***^***d***^
		**p**^**c**^	*0.931*	*0.507*		*0.909*	*0.523*		*0.974*	***0.049***		*0.633*	*0.175*		*0.816*	*0.265*		*0.309*	*0.126*		*0.984*	*0.080*	
CAaN finding in professional life	Yes	31 (% 32.3)	1.70 ± 0.38	4.03 ± 0.44	***<0.001***^**a**^	1.57 ± 0.56	3.93 ± 0.62	***<0.001***^**a**^	1.94 ± 0.46	3.88 ± 0.43	***<0.001***^**a**^	1.94 ± 0.56	3.63 ± 0.48	***<0.001***^**a**^	2.32 ± 0.67	3.38 ± 0.60	***<0.001***^**a**^	2.00 ± 0.63	3.86 ± 0.58	***<0.001***^**a**^	1.87 ± 0.42	3.84 ± 0.38	***<0.001***^**a**^
	No	65 (% 67.7)	1.84 ± 0.53	3.99 ± 0.44	***<0.001***^**a**^	1.68 ± 0.66	3.93 ± 0.56	***<0.001***^**a**^	2.04 ± 0.53	3.86 ± 0.43	***<0.001***^**a**^	2.06 ± 0.58	3.64 ± 0.42	***<0.001***^**a**^	2.52 ± 0.63	3.26 ± 0.50	***<0.001***^**a**^	2.00 ± 0.68	3.78 ± 0.62	***<0.001***^**a**^	1.99 ± 0.49	3.80 ± 0.37	***<0.001***^**a**^
		**p**^**b**^	*0.135*	*0.664*		*0.408*	*0.965*		*0.380*	*0.909*		*0.338*	*0.934*		*0.161*	*0.306*		*0.999*	*0.546*		*0.267*	*0.687*	
CAaN findings in the last year	Yes	8 (% 8.3)	1.80 ± 0.41	3.91 ± 0.47	***0.012***^***d***^	1.68 ± 0.46	3.82 ± 0.72	***0.012***^***d***^	2.09 ± 0.62	3.70 ± 0.30	***0.012***^***d***^	1.94 ± 0.68	3.39 ± 0.52	***0.012***^***d***^	2.33 ± 0.59	3.25 ± 0.55	***0.043***^***d***^	2.06 ± 0.78	3.67 ± 0.48	***0.012***^***d***^	1.96 ± 0.50	3.67 ± 0.36	***0.012***^***d***^
	No	88 (91.7%)	1.79 ± 0.50	4.01 ± 0.44	***<0.001***^**a**^	1.64 ± 0.65	3.94 ± 0.56	***<0.001***^**a**^	2.00 ± 0.50	3.88 ± 0.43	***<0.001***^**a**^	2.02 ± 0.57	3.66 ± 0.42	***<0.001***^**a**^	2.46 ± 0.65	3.30 ± 0.54	***<0.001***^**a**^	2.00 ± 0.65	3.82 ± 0.61	***<0.001***^**a**^	1.95 ± 0.47	3.83 ± 0.37	***<0.001***^**a**^
		**p**^**c**^	*0.780*	*0.533*		*0.599*	*0.581*		*0.633*	*0.184*		*0.647*	***0.039***		*0.554*	*0.617*		*0.790*	*0.410*		*0.942*	*0.230*	
CAaN training at the faculty	Yes	55 (% 57.3)	1.76 ± 0.51	4.02 ± 0.44	***<0.001***^**a**^	1.63 ± 0.68	3.99 ± 0.54	***<0.001***^**a**^	2.05 ± 0.55	3.89 ± 0.41	***<0.001***^**a**^	2.04 ± 0.61	3.71 ± 0.41	***<0.001***^**a**^	2.44 ± 0.57	3.36 ± 0.46	***<0.001***^**a**^	2.03 ± 0.68	3.82 ± 0.59	***<0.001***^**a**^	1.96 ± 0.50	3.85 ± 0.36	***<0.001***^**a**^
	No	41 (% 42.7)	1.84 ± 0.46	3.97 ± 0.43	***<0.001***^**a**^	1.66 ± 0.56	3.85 ± 0.61	***<0.001***^**a**^	1.96 ± 0.44	3.84 ± 0.44	***<0.001***^**a**^	1.99 ± 0.53	3.54 ± 0.46	***<0.001***^**a**^	2.46 ± 0.74	3.22 ± 0.62	***<0.001***^**a**^	1.96 ± 0.64	3.79 ± 0.63	***<0.001***^**a**^	1.95 ± 0.42	3.77 ± 0.37	***<0.001*** ^**a**^
		**p** ^**b**^	*0.446*	*0.597*		*0.850*	*0.241*		*0.429*	*0.633*		*0.645*	*0.054*		*0.876*	*0.227*		*0.606*	*0.825*		*0.914*	*0.271*	
Post-graduation CAaN training	Yes	72 (% 75.0)	1.74 ± 0.44	3.99 ± 0.45	***<0.001*** ^**a**^	1.59 ± 0.60	3.92 ± 0.61	***<0.001*** ^**a**^	1.98 ± 0.49	3.86 ± 0.43	***<0.001*** ^**a**^	1.97 ± 0.54	3.64 ± 0.45	***<0.001*** ^**a**^	2.46 ± 0.65	3.29 ± 0.55	***<0.001*** ^**a**^	1.97 ± 0.64	3.80 ± 0.62	***<0.001*** ^**a**^	1.91 ± 0.43	3.81 ± 0.38	***<0.001*** ^**a**^
	No	24 (% 25.0)	1.97 ± 0.60	4.02 ± 0.40	***<0.001*** ^**a**^	1.80 ± 0.70	3.95 ± 0.48	***<0.001*** ^**a**^	2.11 ± 0.57	3.90 ± 0.41	***<0.001*** ^**a**^	2.15 ± 0.67	3.64 ± 0.39	***<0.001*** ^**a**^	2.42 ± 0.64	3.33 ± 0.51	***<0.001*** ^**a**^	2.10 ± 0.71	3.83 ± 0.56	***<0.001*** ^**a**^	2.07 ± 0.55	3.84 ± 0.33	***<0.001*** ^**a**^
		**p** ^**b**^	***0.047***	*0.784*		*0.154*	*0.873*		*0.251*	*0.646*		*0.250*	*0.973*		*0.763*	*0.730*		*0.392*	*0.818*		*0.202*	*0.738*	
Request to participate in training	Yes	82 (% 85.4)	1.77 ± 0.47	3.98 ± 0.42	***<0.001*** ^**a**^	1.63 ± 0.63	3.90 ± 0.59	***<0.001*** ^**a**^	1.97 ± 0.47	3.85 ± 0.41	***<0.001*** ^**a**^	2.00 ± 0.56	3.62 ± 0.45	***<0.001*** ^**a**^	2.41 ± 0.64	3.31 ± 0.53	***<0.001*** ^**a**^	2.01 ± 0.64	3.81 ± 0.59	***<0.001*** ^**a**^	1.93 ± 0.44	3.80 ± 0.36	***<0.001*** ^**a**^
	No	14 (% 14.6)	1.97 ± 0.57	4.11 ± 0.52	***<0.001*** ^**a**^	1.71 ± 0.65	4.13 ± 0.45	***<0.001*** ^**a**^	2.27 ± 0.66	3.96 ± 0.48	***<0.001*** ^**a**^	2.11 ± 0.66	3.77 ± 0.36	***<0.001*** ^**a**^	2.68 ± 0.68	3.24 ± 0.60	***<0.001*** ^**a**^	1.97 ± 0.77	3.79 ± 0.72	***<0.001*** ^**a**^	2.10 ± 0.59	3.90 ± 0.43	***<0.001*** ^**a**^
		**p** ^**b**^	*0.159*	*0.301*		*0.655*	*0.154*		*0.115*	*0.373*		*0.503*	*0.215*		*0.156*	*0.640*		*0.852*	*0.926*		*0.308*	*0.321*	
Information on study results	Yes	88 (91.7%)	1.75 ± 0.46	3.99 ± 0.43	***<0.001*** ^**a**^	1.61 ± 0.62	3.93 ± 0.59	***<0.001*** ^**a**^	1.97 ± 0.49	3.87 ± 0.42	***<0.001*** ^**a**^	1.98 ± 0.5	3.64 ± 0.44	***<0.001*** ^**a**^	2.39 ± 0.63	3.32 ± 0.52	***<0.001*** ^**a**^	1.98 ± 0.66	3.83 ± 0.59	***<0.001*** ^**a**^	1.91 ± 0.45	3.82 ± 0.36	***<0.001*** ^**a**^
	No	8 (% 8.3)	2.31 ± 0.55	4.11 ± 0.49	***0.012*** ^***d***^	2.04 ± 0.68	3.91 ± 0.32	***0.011*** ^**d**^	2.49 ± 0.42	3.88 ± 0.55	***0.012*** ^***d***^	2.40 ± 0.48	3.64 ± 0.41	***0.012*** ^***d***^	3.13 ± 0.34	3.10 ± 0.72	*0.865*	2.28 ± 0.61	3.53 ± 0.74	***0.012*** ^***d***^	2.41 ± 0.44	3.79 ± 0.47	***0.012*** ^***d***^
		**p** ^**c**^	***0.007***	*0.474*		*0.080*	*0.847*		***0.006***	*0.979*		***0.041***	*0.740*		***0.002***	*0.385*		*0.209*	*0.138*		***0.008***	*0.916*	

n: number, %: percentage rate, a: paired samples t-test, b: independent samples t-test, c: Mann-Whitney U test, d: Wilcoxon signed ranks test. Values with bolded fonts are statistically significant.

The same situation applies to the BS subcategory mean score for participants who do not have children and who do not give particular importance to signs of neglect and abuse in children brought in for examination, and to the PC subcategory mean score for those who stated that they had encountered signs of neglect and/ or abuse in the last year. However, as seen in Table 5, while the differences between the subcategory and scale mean scores were significant before the training, this situation disappeared in the post-test.

**Figure 1:**
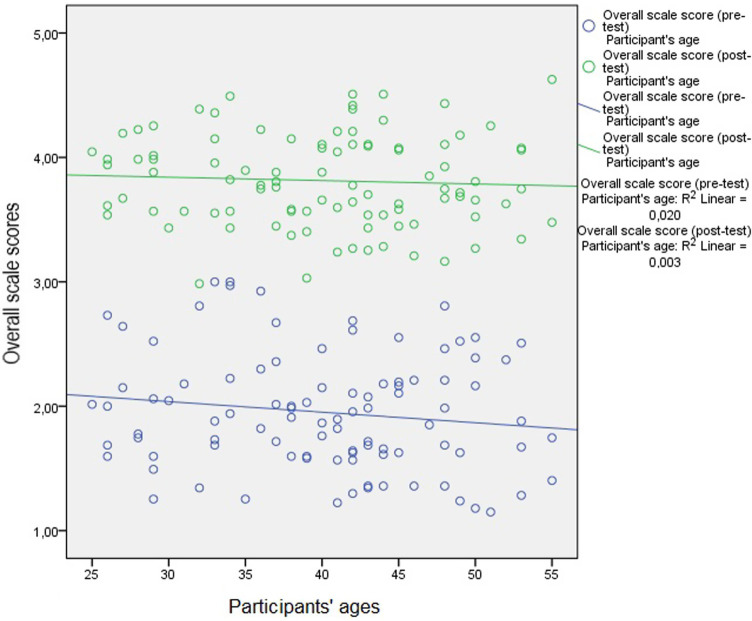
Relationship between participants’ ages and pre-test and post-test scale score averages.

**Figure 2:**
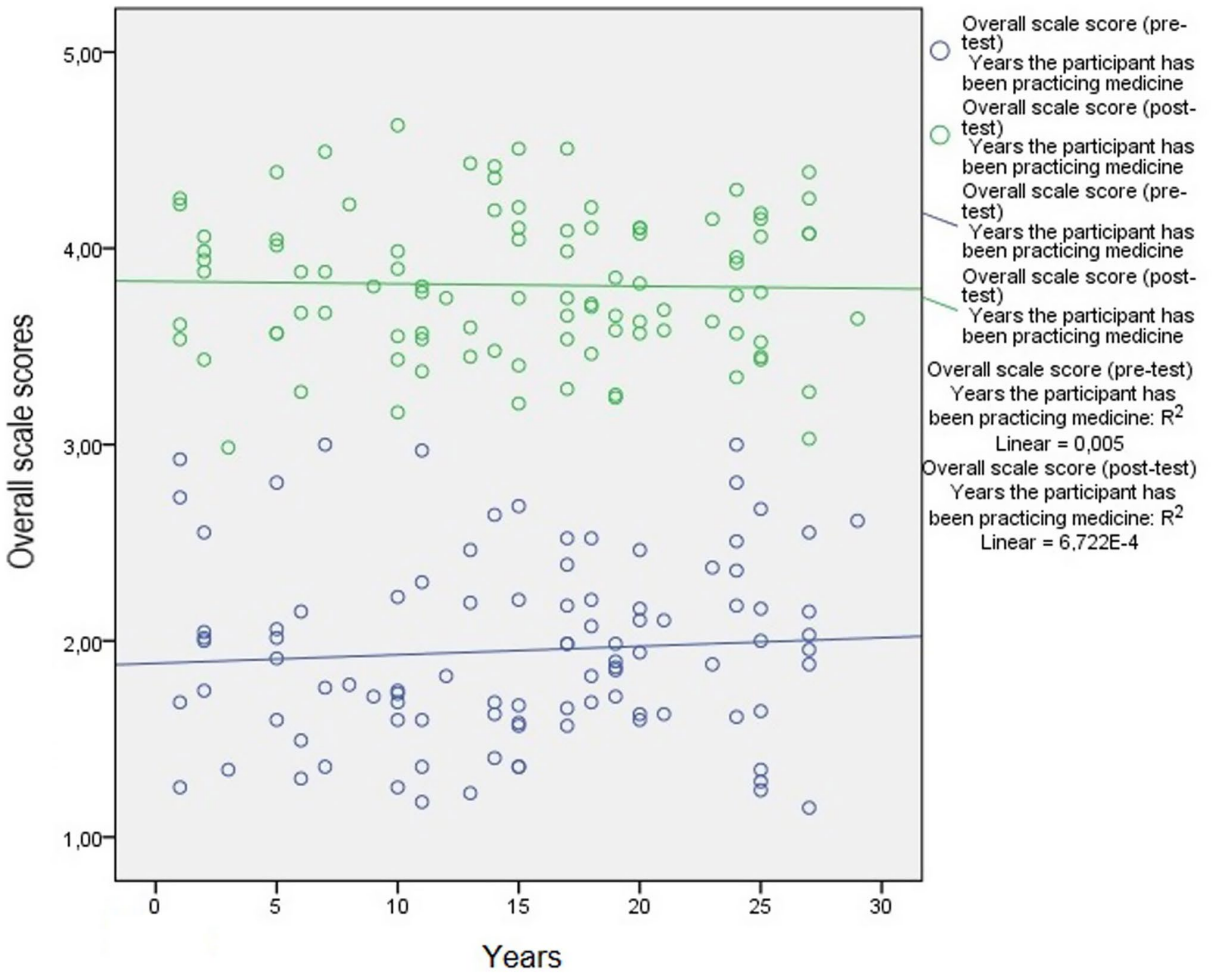
The relationship between the years the participants have been practicing medicine and the pre-test and post-test scale score averages.

The weak level of relationship, which can be seen in Figures 1, 2, was found to be statistically insignificant (*p* > 0.05) as demonstrated by the results of the correlation analysis performed to examine the relationship between the participants’ ages and the length of time they have been practicing medicine and the pre-test and post-test scale score averages.

## 4 Discussion

Child abuse is almost always with child neglect in forms of emotional, physical, and sexual abuse, and according to WHO data, at the beginning of the 21st century unfortunately, one-fourth of adults, including one-thirteenth of men and a fifth of women, were abused sexually in childhood (13).

Physical abuse, the most prevalent symptom of which is skin lesions in children; bite marks, abrasions, bruising, burn findings, and sharp object trauma, poisoning and asphyxia should definitely be considered when they cannot be explained by parents for another reason and sufficiently (14, 15). Also, the orofacial region’s detailed clinical observation and recording of findings are essential for spotting certain patterns of physical abuse (16, 17). Emotional abuse is an important phenomenon for children who are not given enough care, love, and attention. Children who exhibit unexpected sexual behaviors for their age, sexually transmitted disease findings, and genital bleeding symptoms should also be evaluated for sexual abuse. Non-specific complaints such as enuresis, encopresis, and sleep disorders are also important as the prominent signs in children who were abused sexually (18, 19). Sexual abuse, like other types of abuse, has a several number of symptoms including lesions of the oral cavity and can result in eating disorders, memory loss, depression, aggression, and anger (20). Neglected children manifest symptoms of aggression in their future lives and have different kinds of problems emotionally such as self-confidence and learning problems. It is noted that the lack of basic care needs of the child, such as education, cleanliness, nutrition, and health, should be considered a sign of the child’s neglect (17).

Although family physcians are not the only group of health professionals who can be the front line in CAaN situations, it is possible to detect all these signs and symptoms at an early stage in family health centers because one of the most basic tasks performed in these centers is to monitor adolescents, children, and infants (21). For this reason, it is stated that family health centers, which are the first point of application in the health system, can also be considered an important gatekeeper for preventing CAaN (6).

As family physicians, medical doctors working in these centers, to whom such an important responsibility is attributed; the subcategories in which they received the highest average scores in the pre-test applied at the beginning of our study are as follows: CC subcategory (2.45 ± 0.65), PC subcategory (2.02 ± 0.57), BS subcategory (2.01 ± 0.51), FC subcategory (2.00 ± 0.66), PS subcategory (1.80 ± 0.50) and SN subcategory (1.64 ± 0.63). The general scale score average of the pre-test is (1.95 ± 0.47). This situation reflects that their theoretical knowledge of the subject, although not at a sufficient level, is better than their knowledge level of findings that can be detected by physical examination.

According to the post-test conducted following the in-service training provided by academicians who have studies on the subject, the participants’ sub-category mean scores, from highest to lowest are; PS subcategory (4.00 ± 0.44), SN subcategory (3.93 ± 0.57), BS subcategory (3.87 ± 0.42), FC subcategory (3.81 ± 0.60), PC subcategory (3.64 ± 0.44) and CC subcategory (3.30 ± 0.54). The post-test general scale mean score is (3.81 ± 0.37). In other words, after the training, both the general scale mean score and the sub-category mean scores increased statistically significantly, reaching a level that reflects that they have sufficient knowledge of the subject. In other words, in-service training has a visible positive effect, just like the indirect training received during undergraduate education programs (22–24).

In a study conducted with teachers who are employees of a different sector, the vast majority of participants stated that they would not make a legal report if they detected child neglect and abuse (25). In our study, when we look at the reasons for hesitation in reporting cases of child neglect and abuse, it is striking that these reasons are; the belief that the pertinent officialdom will not take necessary actions is thought by two-thirds of the participants. In the study conducted with family physicians in the province of Mersin, the same reason was also put forward by almost half of the participants there (6). While the fear of being worn out, pressured, and threatened in processes such as police stations, courts, etc. is another prominent reason for hesitation, not knowing whether or how to report and not wanting to be involved in an unpleasant issue were expressed as other reasons at the same level. The belief that the child’s life will be affected in a way that is not desirable following the report and not suspecting neglect and abuse are the reasons put forward by approximately one-third of the participants. The proportional distribution of the reasons for hesitation is consistent with the study in question and the study conducted in the east of Turkey on the same subject (6, 26). This situation indicates that health professionals should be informed through appropriate training about the notification process and legal process after the detection of a negative situation.

A recent scoping review, which included 10 separate studies with doctors from Australia, Canada, the United Kingdom, the United States of America and New Zealand, who worked in a variety of specialties including pediatricians, forensic physicians, gynecologists and ophthalmologists, highlights the range of impacts experienced by doctors who are called to court or testify in child abuse cases, as well as the lack of measures aimed at mitigating negative personal and professional outcomes. It noted that there is an urgent need for increased education and training, policy and practice reforms, and research into interventions that could potentially mitigate these negative outcomes in order to assist doctors more effectively as expert witnesses (27).

In a study conducted with pediatric surgeons, two-thirds of the participants stated that their knowledge on this subject was inadequate (28), while in a study conducted with health professionals working in private hospitals in Istanbul, half of the participating doctors reported that they had difficulty with anamnesis taking and legal processes (29). In a study conducted with family physicians working in Erzurum, one-third of the participants thought they were weak in obtaining information about medical history in these situations (30). In our study, the topics that most participants wanted to include in the training to be organized are consistent with the situations stated in these studies. Also, in a study conducted with pediatricians and pedodontists working throughout Turkey, the obstacles stated by the participants in the management of abuse cases were similar (31).

Another scoping review, which analyzed a total of 10 studies that provided a synthesis of the existing literature on medical interviewing practices in CAaN cases, noted a significant gap in knowledge regarding effective information gathering techniques for CAaN in healthcare settings. Studies examined a variety of forms of abuse encountered by medical professionals and nursing staff, including child sexual abuse, physical abuse, and neglect. In particular, forensic settings and emergency departments were highlighted as key healthcare contexts in which these issues occur. Findings suggest that although healthcare practitioners often collect information through the medical history of affected children, there is a lack of standardized guidelines for specific questions to ask. An interesting point in this review is that no studies were found that focused on the information gathering competencies of oral health practitioners (32).

## 5 Limitations of the study

However, the current study has limitations that should be considered when interpreting the results. First, the sample is limited to one district of the country, which may affect the generalizability of the findings to all family physicians. Additionally, personal experiences and opinions potentially impacting their knowledge, attitudes, and perceptions could have influenced participant responses. Failure to account for these limitations may impede the generalizability of the study’s findings.

## 6 Conclusion and recommendations

It is an indisputable fact that the detection of CAaN by all health professionals, especially doctors, without exception is important from both professional, legal, and humanitarian perspectives. The role of family physicians, who periodically evaluate people in this vulnerable group due to routine follow-ups, even if they do not have any illness, in early detection and reporting is becoming even more important.

The results of this study reveal that in-service training given to family physicians has positive effects on the detection of CAaN. Therefore, awareness and knowledge levels regarding this sensitive issue should be maintained with regular in-service trainings. In addition, the processes that enable them to fulfill their legal obligations should also be facilitated. Additional security should be provided to family physicians who make reports by ensuring that their information is anonymous.

## Data Availability

The raw data supporting the conclusions of this article will be made available by the authors, without undue reservation.
